# Association of early life factors and acute lymphoblastic leukaemia in childhood: historical cohort study

**DOI:** 10.1038/sj.bjc.6600012

**Published:** 2002-02-01

**Authors:** L Murray, P McCarron, K Bailie, R Middleton, G Davey Smith, S Dempsey, A McCarthy, A Gavin

**Affiliations:** Northern Ireland Cancer Registry, Department of Epidemiology and Public Health, The Queens University, Belfast, Riddel Hall, Stranmillis Road, Belfast BT9 5EE, UK; Division of Cancer Control and Population Sciences, National Cancer Institute, Rockville, Maryland, USA; Ulster Community & Hospitals Trust, Belfast, UK; Department of Social Medicine, University of Bristol, Bristol, UK; Royal Group of Hospitals Trust, Belfast, UK

**Keywords:** ALL, childhood, early life factors, historical cohort

## Abstract

In a historical cohort study of all singleton live births in Northern Ireland from 1971–86 (*n*=434 933) associations between early life factors and childhood acute lymphoblastic leukaemia were investigated. Multivariable analyses showed a positive association between high paternal age (⩾35 years) and acute lymphoblastic leukaemia (relative risk=1.49; 95% confidence interval (CI)=0.96–2.31) but no association with maternal age. High birth weight (⩾3500 g) was positively associated with acute lymphoblastic leukaemia (relative risk=1.66; 95% CI=1.18–2.33). Children of mothers with a previous miscarriage or increased gestation (⩾40 weeks) had reduced risks of ALL (respective relative risks=0.49; 95% CI=0.29–0.80, and 0.67; 95% CI=0.48–0.94). Children born into more crowded households (⩾1 person per room) had substantially lower risks than children born into less crowded homes with also some evidence of a lower risk for children born into homes with three adults (relative risks=0.56; 95% CI=0.35–0.91 and 0.58; 95% CI=0.21–1.61 respectively). These findings indicate that several early life factors, including living conditions in childhood and maternal miscarriage history, influence risk of acute lymphoblastic leukaemia in childhood.

*British Journal of Cancer* (2002) **86**, 356–361. DOI: 10.1038/sj/bjc/6600012 www.bjcancer.com

© 2002 The Cancer Research Campaign

## 

The aetiology of childhood leukaemia, the most common childhood malignancy, is poorly understood and there are few established risk factors. Recently there has been growing interest in the relationship between prenatal and early life influences on risk of childhood leukaemia (Van Steensel-Moll *et al*, 1985; Shu *et al*, 1988; Kaye *et al*, 1991; Westergaard *et al*, 1997; McKinney *et al*, 1999). It is clear that mutations occurring in-utero in lymphoid stem cells contribute to the development of childhood leukaemia (Greaves, 1999; Wiemels *et al*, 1999) but it is not known which prenatal exposures result in these mutations or how they may be prevented. The apparent association between high birth weight and increased risk of acute lymphoblastic leukaemia (ALL) has stimulated research into the role of insulin growth factor-1 (IGF-1) and relevant receptors in abnormal haematopoiesis. Other possible prenatal or early life influences, as indexed, for example, by birth order or social class at birth have less well understood potential mechanisms. Evidence from studies on population mixing (Kinlen, 1995) indicate a role for infectious agents and delayed exposure to infection has been suggested as influencing risk of childhood leukaemia (Greaves and Alexander, 1993; Greaves, 1997).

There is a lack of consistency among reports examining prenatal and early life associations with childhood leukaemia, suggesting that some of the observed associations are spurious. Study designs utilized to date may have contributed to this lack of consistency and very few population-based cohort studies of childhood leukaemia have been performed (Westergaard *et al*, 1997; Mogren *et al*, 1999). We describe a population-based cohort study, undertaken in Northern Ireland, which includes unique information recorded at birth on socio-economic position and living conditions in the household.

## MATERIALS AND METHODS

Since 1971, the Northern Ireland Child Health System has collected information on all births to mothers normally resident in Northern Ireland. The Department of Health, Social Services and Public Safety maintains the data relating to births between 1971 and 1986. Subject to an appropriate confidentiality agreement, access was granted to these data which include: date and place of birth; birth weight (in grams); gestational age (to the nearest complete week); number of previous pregnancies, live births and miscarriages; mother's and father's year of birth; social class (based on father's occupation); method of infant feeding on discharge from place of confinement; number of persons aged above and below 15 years of age per household and; number of living rooms and bedrooms in the household at the time of the birth.

Details of incident cases of leukaemia which occurred before the age of 16 years in Northern Ireland between 1975 and 1997 were obtained from the Northern Ireland Cancer Registry's Register of Childhood Cancers. Data on cases are obtained from multiple sources, including: the Northern Ireland Leukaemia and Lymphoma Register; the Oxford Childhood Cancer Register; clinicians' records and; databases of specific research projects into childhood cancer. Using sex, surname, and date of birth, cases of ALL born in Northern Ireland between 1971 and 1986 were identified within the Child Health System database. Exact matches, and matches where one character was incorrect were accepted after manual checking. Perinatal information relating to the children who subsequently developed ALL was compared to that of children who did not develop ALL. Multiple births were excluded from the analyses. There were too few cases of acute myeloid leukaemia (*n*=27) to permit investigation in the same manner.

### Univariable analyses

Approximate maternal and paternal age, at time of the child's birth, were calculated by subtracting mother's and father's year of birth from year of birth of the child. Household density was calculated as the number of residents of all ages (including the newborn) divided by the number of rooms in the accommodation. These variables and number of previous miscarriages, birth order, diagnosis of Down's syndrome at birth, method of feeding, and number of children and adults in the household were categorized as in Table 1

**Table 1:**
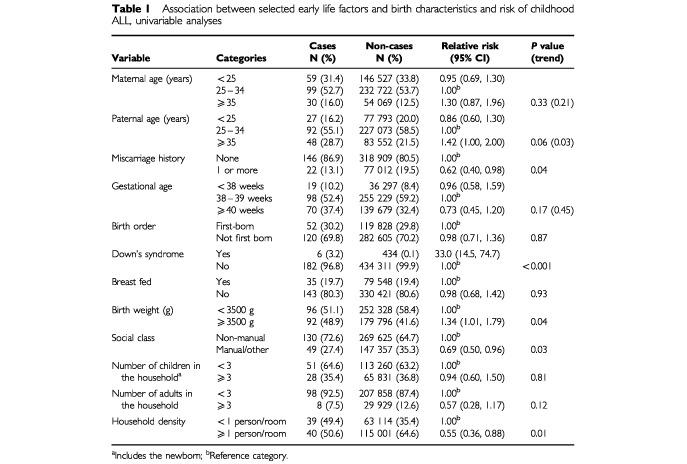
Association between selected early life factors and birth characteristics and risk of childhood ALL, univariable analyses

. Social class, based on father's occupation, was coded in the Child Health System according to the Office of Population Censuses and Surveys classification of occupations into six groups as categorized in Table 2

**Table 2:**
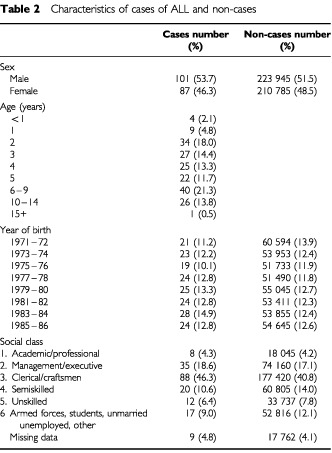
Characteristics of cases of ALL and non-cases

. In analyses this was dichotomized into non-manual (groups 1–3) and manual/other (groups 4–6) occupations. Differences in proportions in these predictor variables were compared between those children who did and those who did not develop ALL using the χ^2^ test and the χ^ 2^ test for trend. Where appropriate, variables were dichotomized and χ^2^ tests repeated. Parental age was dichotomized into <35 years and ⩾35 years, and gestational age into <40 weeks and ⩾40 weeks.

### Multivariable analyses

As we did not actively follow up the cohort for migration or death we did not feel justified in calculating person years and modelling the risk of disease using Poisson or Cox regression. Instead we approximated relative risks by the odds ratios obtained from logistic regression models, an approximation which is reasonable for a rare disease such as childhood ALL. Variables with a *P*<0.2 level in univariable analyses were included in multivariable models. As data relating to living conditions were unavailable before 1976 or after 1984, two multivariable models were constructed: model I did not include number of adults in the household or household density; model II included these variables. These models were also adjusted for sex and Down's syndrome and models were also constructed to include only those children with a diagnosis of ALL before the age of 6 years. All statistical analyses were performed using SPSS for Windows, Version 10.0 (SPSS Inc.,Chicago, USA). Ethical approval was obtained from the Research Ethics Committee of The Queen's University of Belfast.

## RESULTS

The Child Health System contained data on 444 168 live births (including 9235 multiple births) which occurred between January 1971 and December 1986 to mothers normally resident in Northern Ireland. The Registrar General's Office, which registers all births within Northern Ireland, registered 444 111 live births for the same period. The Child Health System may include a small number of duplicate records. The Northern Ireland Cancer Registry contains 342 cases of ALL diagnosed in children aged less than 16 years in Northern Ireland between January 1975 and December 1997. Of these, 212 were born between 1971 and 1986, and 189 (89.2%) were identified within the Child Health System. One was a multiple birth and was excluded from the analyses. This study therefore involves 188 cases of childhood ALL and 434 745 non cases. Characteristics of the identified cases are shown in Table 2. The male to female ratios of identified cases and unidentified cases were 1 : 1.16 and 1 : 1.09 respectively. Mean age at diagnosis in identified and unidentified cases was 5.19 and 5.26 years respectively, *t*-test value 0.1, *P*=0.92.

### Univariable analyses

Table 1 shows the association between variables of interest and the risk of childhood ALL. There were 446 children who had a diagnosis of Down's syndrome made at birth, six of whom subsequently developed ALL (relative risk=33.0; 95% CI=14.5–74.7). High paternal and maternal age (35 years) were both associated with a modest increase in risk of ALL while the risk associated with young parental age (<25 years), especially in mothers, did not differ substantially from that of parental age between 25 and 34 years. When dichotomized into <35 and ⩾35 years the relative risk associated with high paternal age was 1.47 (95% CI=1.05–2.06; *P*=0.02), while that associated with high maternal age was 1.33 (95% CI=0.90–1.97; *P*=0.15). Women who had at least one miscarriage were substantially less likely to have a child who developed ALL than women who had no history of miscarriage (relative risk=0.62; 95% CI=0.40–0.98). Gestation of more than 40 weeks appeared to be protective against future risk of ALL while the risk associated with gestation of less than 38 weeks was similar to that for 38–39 weeks. When gestational age was dichotomized into <40 weeks or ⩾40 weeks, longer gestation had a relative risk of 0.76 (95% CI=0.57–1.01; *P*=0.06). Higher birth weight (⩾3500 g) was associated with a modest increase in risk of ALL (relative risk=1.34; 95% CI=1.01–1.79). There was no association between risk of ALL and birth order or breast-feeding on discharge from place of confinement. The strongest associations seen were for social class at birth and living conditions in the household into which the child was born. Children whose fathers were in manual occupations at the time of birth had a risk of ALL more than 30% lower than children whose fathers were employed in non-manual occupations. Although number of children in the household at the time of birth was unrelated to risk of ALL, children born into households in which three or more adults were living appeared to have substantially reduced risk of ALL (relative risk=0.57; 95% CI=0.28, 1.17). Furthermore, being born into a more crowded home was associated with a 45% reduction in risk of ALL.

### Multivariable analyses

Tables 3

**Table 3:**
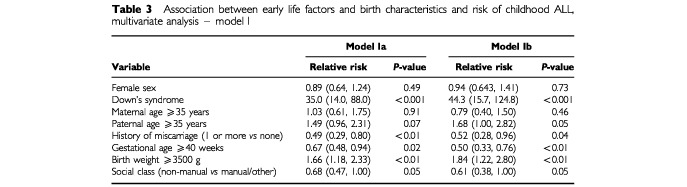
Association between early life factors and birth characteristics and risk of childhood ALL, multivariate analysis – model I

and 4

**Table 4:**
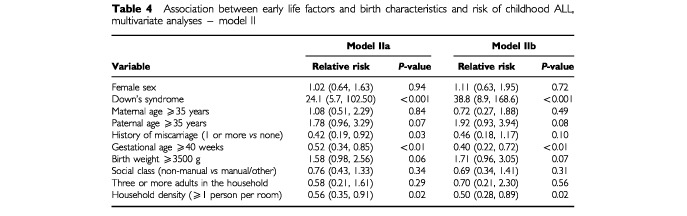
Association between early life factors and birth characteristics and risk of childhood ALL, multivariate analyses – model II

show the mutually adjusted relative risks for the associations between predictor variables and risk of ALL. Not all data items were available for every subject. Model Ia (Table 3) is based on 146 cases (77.7% of all cases) and 345 473 children who did not develop ALL (79.5% of total). Model IIa (Table 4) is based on 72 children who developed ALL and 150 772 who did not; 66.7 and 62.9%, respectively, of those born in the years during which data on living conditions were collected. In Model Ia high maternal age was no longer associated with risk of ALL, although high paternal age continued to be associated with a 50% increase in risk. However, maternal and paternal age were closely correlated; correlation co-efficient 0.81, *P*<0.001. The remaining adjusted risks in Model Ia were either unaltered or stronger than the unadjusted risks.

Further adjustment for number of adults in the household and household density at birth (Model IIa) again strengthened the associations between paternal age, history of miscarriage, and gestational age, and risk of ALL. In this model, paternal age of 35 years or over was associated with an 80% increase in the risk of ALL, while history of miscarriage and longer gestational age were associated with 60 and 50% lower risks, respectively. The negative association between social class and ALL was attenuated while that for birth weight was unaltered. The strong association between household density and risk of childhood ALL seen in the univariable analysis remained in this multivariable model, with children born into less crowded households having almost twice the risk of ALL compared to children from more crowded households.

Restricting the multivariate models to only those cases diagnosed before 6 years (Models Ib and IIb, Tables 3 and 4 respectively) either strengthened or did not alter the relative risks observed in the models including all cases. Maternal age was the exception – the direction of the relative risk observed changed from weakly positive to weakly negative between Models Ia and Ib, and between Models IIa and IIb.

## DISCUSSION

A major strength of this study is that it involved follow-up of a whole population for virtually the full period of risk of childhood ALL. As far as we are aware it is only the third such population-based cohort study of childhood ALL and it is unique in that the data, collected at the time of birth, included information on childhood socioeconomic circumstances and household living conditions. The use of several different sources of data to identify incident cases of ALL in a relatively small geographic region, where few centres treat this condition will have resulted in a very high level of case ascertainment. Selection bias through inclusion of only those cases treated at specific centers has been avoided. Non-inclusion of cases was uncommon and was due only to failure to identify their birth records, which with the exception of children moving into Northern Ireland was likely to be entirely random. The data used in the analyses were documented before the onset of illness, thus avoiding recall bias and observer bias. Inclusion of all live births also avoids the biases associated with control selection and participation in case-control studies. Furthermore, as the Northern Irish population is ethnically homogenous, racial and genetic factors which may obscure important associations in more mixed populations may be discounted.

The study has some potential limitations. The relatively small number of cases precludes important subgroup analyses. Birth weight was the only available indicator of prenatal development, and data on living conditions within the household were not collected for the whole study period. Data were collected by many observers and, although attempts were made to standardize procedures, variation in recording is likely to have occurred. However, such misclassification bias will result in the underestimation of true associations. The matching process will not have identified ALL cases born outside marriage, who were initially given their mother's surname and had a subsequent name change such as when their mother married. As unmarried mothers are more likely to be younger and live with relatives (higher household densities) than their married counterparts, failure to identify these cases will have reduced the power of the study to detect associations between household density, or young maternal age, and ALL. However, as birth records were identified for almost 90% of possible cases, this is a minor deficiency.

### Birth weight

We found a reasonably strong positive association between birth weight and risk of childhood ALL. To date, the findings on birth weight and risk of childhood leukaemia have been inconsistent. Several studies have reported positive relationships with ALL (Robison *et al*, 1987; Kaye *et al*, 1991; Westergaard *et al*, 1997) while others (McKinney *et al*, 1987, 1999; Roman *et al*, 1997; Dockerty *et al*, 1999), found no association or a relationship with both high and low birth weight (Schuz *et al*, 1999). It is possible that selection bias, particularly among controls, may have resulted in the failure of some investigators to observe a positive association as controls are likely to be of higher socio-economic position and thus have infants of higher average birth weight, than the general population (Kogan, 1995). Our study did not have this problem and multivariable analyses suggest that differences in birth weight across social classes do not explain our observation. Choice of threshold for high birth weight may also contribute to the inconsistency between reports. We found that the relationship between high birth weight and risk of ALL was much less obvious if a threshold of 4000 g rather than 3500 g was chosen and at least one other group has found the threshold employed to be crucial (Kaye *et al*, 1991). This sensitivity to the choice of threshold may suggest that the association is spurious. On the other hand, we displayed the same relationship, and a similar magnitude of effect, as one other population-based cohort (Westergaard *et al*, 1997). The relationship is also biologically plausible and evidence is accumulating to suggest that it may be mediated through IGF-1 (Ross *et al*, 1996; Yeazel *et al*, 1997; Petridou *et al*, 1999; Blatt, 2000; Eshet *et al*, 2000).

### Parental age

Although higher maternal age has generally been associated with higher risk of ALL (Kaye *et al*, 1991; Dockerty *et al*, 1999; Hemminki *et al*, 1999; Mogren *et al*, 1999), not all studies have observed this relationship (Shaw *et al*, 1984; Van Steensel-Moll *et al*, 1985; Shu *et al*, 1988; Westergaard *et al*, 1997; Rosenbaum *et al*, 2000) and one has demonstrated higher risk at younger maternal age (Schuz *et al*, 1999). We did find any association with maternal age. Paternal age has been infrequently examined as a risk factor for childhood ALL, with reports of both a positive association (Kaye *et al*, 1991) and no association (Shaw *et al*, 1984; Westergaard *et al*, 1997; Hemminki *et al*, 1999). We demonstrated a strong relationship between ALL and paternal age of 35 years or more. However, paternal age is highly correlated with maternal age and inclusion of the two variables in the same model may have an unpredictable effect on observed relative risks. In our univariable analyses paternal age was more strongly related to risk of ALL than was maternal age, indicating that higher paternal age may be a risk factor for childhood ALL independently of maternal age.

### Gestational age and previous history of miscarriage

We found gestation beyond 40 weeks to be associated with a substantial decrease in the risk of childhood ALL. Most previous reports failed to find an association between ALL and length of gestation (Kaye *et al*, 1991; Kaatsch *et al*, 1996; McKinney *et al*, 1999), although one study has shown a similar, but not statistically significant, relationship (Dockerty *et al*, 1999). Some studies have reported positive associations between history of previous foetal loss and childhood leukaemia (Van Steensel-Moll *et al*, 1985; Kaye *et al*, 1991), others found no association (Schuz *et al*, 1999) while in the current study, as in a Chinese case–control study (Shu *et al*, 1988) an inverse association was found. Possible mechanisms for a positive association include environmental exposures and genetic predisposition but the issues of selective participation and recall bias have not been fully addressed. As our findings are free from such biases they may therefore reflect the true relationship between prior history of miscarriage and risk of childhood ALL.

### Socioeconomic status and household density

As in this study, some investigators have shown that higher socio-economic position, is associated with higher risk of ALL (McWhirter, 1982; Kaye *et al*, 1991; Dockerty *et al*, 1999) but others have failed to observe this association (Shaw *et al*, 1984; Van Steensel-Moll *et al*, 1985, 1986; Shu *et al*, 1988; Kaatsch *et al*, 1996; McKinney *et al*, 1999; Rosenbaum *et al*, 2000) and some have found the opposite pattern, both for childhood ALL (Brondum *et al*, 1999; Dockerty *et al*, 1999) and infant leukaemia (Ross *et al*, 1997). In some of these studies subjects from higher socio-economic and better educational backgrounds are overrepresented among controls, reducing the ability to detect a positive association between socio-economic position and childhood ALL. This was not a problem in the current study and the availability of data on social class recorded at the time of birth adds to the robustness of our findings. The positive relationship with social class is further supported by the strong inverse association we demonstrated between household density and risk of childhood ALL. This finding is in keeping with the inverse (although not statistically significant), relationship between household density and childhood ALL which was observed in a case control study from New Zealand (Dockerty *et al*, 1999). However, as far was we are aware, this is the first time the association has been demonstrated in a cohort study and our findings are likely to reflect the true relationship between socio-economic position, household density and risk of childhood ALL.

Our finding of around a two-fold greater risk of childhood ALL among children born into surroundings where there is little overcrowding supports the hypothesis that childhood ALL is caused by an inappropriate immunological response to a common infection or infections in children whose immune systems are not ‘programmed’ by early exposure to these infections (Greaves and Alexander, 1993; Greaves, 1997). In support of this hypothesis, several studies have reported that early exposure to infection (Van Steensel-Moll *et al*, 1986; Kaatsch *et al*, 1996; McKinney *et al*, 1999) is associated with a reduced risk of childhood ALL. However, contrary to what may be expected if this hypothesis is correct, we and several other investigators (Shaw *et al*, 1984; Shu *et al*, 1988; Kaye *et al*, 1991; McKinney *et al*, 1999; Schuz *et al*, 1999; Rosenbaum *et al*, 2000) did not find that later-born infants had a lower risk of ALL than first-born infants. Also we did not demonstrate a relationship between risk of ALL and number of children in the household, while being born into a household containing three or more adults appeared to be protective. If early exposure to infections in childhood protects against ALL, transmission of infections from adults to children may be more important.

## CONCLUSION

This population based cohort study is the first such study to demonstrate that being born into a household of high socio-economic position or low household density is a risk factor for the development of childhood ALL. There is also evidence that being born into an extended family may be protective. These findings offer support for the theory that modulation of the immune system by early exposure to infection is important in reducing future risk of ALL. Further investigation of this hypothesis is warranted, including the examination of infections that are likely to be transmitted, within households, from adults to children.

## References

[bib1] BlattJ2000 IGF1 and Leukemia Pediatr Hematol Oncol 17 199201 10779985 10.1080/088800100276361

[bib2] BrondumJShuXOSteinbuchMSeversonRKPotterJDRobisonLL1999 Parental cigarette smoking and the risk of acute leukemia in children Cancer 85 13801388 10189146

[bib3] DockertyJDSkeggDCElwoodJMHerbisonGPBecroftDMLewisME1999 Infections, vaccinations, and the risk of childhood leukemia Br J Cancer 80 14831489 10424755 10.1038/sj.bjc.6690548 PMC2363060

[bib4] EshetRZaizovRFreudELaronZ2000 Decreased insulin-like growth factor-1 receptor sites on circulating mononuclear cells from children with acute leukemia Pediatr Hematol Oncol 17 253260 10779992 10.1080/088800100276433

[bib5] GreavesMFAlexanderFE1993 An infectious aetiology for common acute lymphoblastic leukemia in childhood? Leukemia 7 349360 8445941

[bib6] GreavesMF1997 Aetiology of acute leukemia Lancet 349 344349 9024390 10.1016/s0140-6736(96)09412-3

[bib7] GreavesM1999 Molecular genetics, natural history and the demise of childhood leukemia Eur J Cancer Prev 35 173185 10.1016/s0959-8049(98)00433-x 10448256

[bib8] HemminkiKKyyronenPVaitinenP1999 Parental age as a risk factor of childhood leukemia and brain cancer in offpring Epidemiology 10 271275 10230837

[bib9] KaatschPKaletchUKrummenauerFMeinertRMiesnerAHaafGMichaelisJ1996 Case-control study on childhood leukemia in Lower Saxony, Germany. Basic considerations, methodology and summary of results Klin Paediatr 208 179185 10.1055/s-2008-1046470 8776704

[bib10] KayeSARobisonLLSmithsonWAGundersonPKingFLNegliaJP1991 Maternal reproductive history and birth characteristics in childhood acute lymphoblastic leukemia Cancer 68 13511355 1873786 10.1002/1097-0142(19910915)68:6<1351::aid-cncr2820680627>3.0.co;2-j

[bib11] KinlenLJ1995 Epidemiological evidence for an infective basis in childhood leukemia Br J Cancer 71 15 7819022 10.1038/bjc.1995.1 PMC2033475

[bib12] KoganMD1995 Social causes of low birth weight J R Soc Med 88 611615 8544143 10.1177/014107689508801103 PMC1295382

[bib13] McKinneyPACartwrightRASaiuJMMannJRStillerCADraperGJHartleyALHoptonPABirchJMWaterhouseJA1987 The inter-regional epidemiological study of childhood cancer (IRESCC): a case-control study of aetiological factors in leukemia and lymphoma Arch Dis Child 62 279287 3646026 10.1136/adc.62.3.279 PMC1778298

[bib14] McKinneyPAJuszczakEFindlayESmithKThomsonCS1999 Pre- and perinatal risk factors for childhood leukemia and other malignancies: a Scottish case control study Br J Cancer 80 18441851 10468308 10.1038/sj.bjc.6690609 PMC2374272

[bib15] McWhirterWR1982 The relationship of incidence of childhood lymphoblastic leukemia to social class Br J Cancer 46 640645 6958309 10.1038/bjc.1982.249 PMC2011178

[bib16] MogrenIDamberLTavelinBHogbergU1999 Characteristics of pregnancy and birth and malignancy in the offspring (Sweden) Cancer Causes Control 10 8594 10334647 10.1023/a:1008813701634

[bib17] PetridouEDessyprisNSpanosEMantzorosCSkalkidouAKalmantiMKoliouskasDKosmidisHPanagiotouJPPiperopoulouFTzortatouFTrichopoulosD1999 Insulin-like growth factor-I and binding protein-3 in relation to childhood leukemia Int J Cancer 80 494496 9935146 10.1002/(sici)1097-0215(19990209)80:4<494::aid-ijc2>3.0.co;2-k

[bib18] RobisonLLCoddMGundersonPNegliaJPSmithsonWAKingFL1987 Birth weight as a risk factor for acute lymphoblastic leukemia Pediatr Hematol Oncol 4 6372 3152913 10.3109/08880018709141250

[bib19] RomanEAnsellPBullD1997 Leukemia and non-Hodgkin's lymphoma in children and young adults: are prenatal and neonatal factors important determinants of disease Br J Cancer 76 406415 9252212 10.1038/bjc.1997.399 PMC2224068

[bib20] RosenbaumPFBuckGMBrecherML2000 Early child-care and preschool experiences and risk of childhood acute lymphoblastic leukemia Am J Epidemiol 152 11361144 11130619 10.1093/aje/152.12.1136

[bib21] RossJAPerentesisJPRobisonLLDaviesSM1996 Big babies and infant leukemia: a role for insulin-like growth factor-1? Cancer Causes Control 7 553559 8877054 10.1007/BF00051889

[bib22] RossJAPotterJDShuXOReamanGHLampkinBRobisonLL1997 Evaluating the relationships among maternal reproductive history, birth characteristics, and infant leukemia – a report from the Children's Cancer Group Ann Epidemiol 7 172179 9141639 10.1016/s1047-2797(97)00012-4

[bib23] SchuzJKaatchPMeinertRMichaelisJ1999 Association of childhood cancer with factors related to pregnancy and birth Int J Epidemiol 28 631639 10480689 10.1093/ije/28.4.631

[bib24] ShawGLaveyRJacksonRAustinD1984 Association of childhood leukemia with maternal age, birth order, and paternal occupation Am J Epidemiol 119 788795 6720675 10.1093/oxfordjournals.aje.a113799

[bib25] ShuXOGaoYTBrintonLALinetMSTuJTZhengWFraumeniJF1988 A population-based case-control study of childhood leukemia in Shanghai Cancer 62 635644 3164642 10.1002/1097-0142(19880801)62:3<635::aid-cncr2820620332>3.0.co;2-3

[bib26] Van Steensel-MollHAValkenburgHAVandenbrouckeJPvan ZanenGE1985 Are maternal fertility problems related to childhood leukemia Int J Epidemiol 14 555559 3866751 10.1093/ije/14.4.555

[bib27] Van Steensel-MollHAValkenburgHAvan ZanenGE1986 Childhood leukemia and infectious diseases in the first year of life: a register-based case-control study Am J Epidemiol 124 590594 3463201 10.1093/oxfordjournals.aje.a114431

[bib28] WestergaardTAndersenPKPedersenJBOlsenJHFrischMSorensenHTWohlfahrtJMelbyeM1997 Birth characteristics, sibling patterns, and acute leukemia risk in childhood: a population-based cohort study J Natl Cancer Inst 89 939947 9214673 10.1093/jnci/89.13.939

[bib29] WiemelsJLCazzanigaGDaniottiMEdenOBAddisonGMMaseraGSahaVBiondiAGreavesMF1999 Prenatal origin of acute lymphoblastic leukemia in children Lancet 354 14991503 10551495 10.1016/s0140-6736(99)09403-9

[bib30] YeazelMWRossJABuckleyJDWoodsWGRuccioneKRobisonLL1997 High birth weight and risk of specific childhood cancers: a report from the Children's Cancer Group J Pediatr 131 671677 9403644 10.1016/s0022-3476(97)70091-x

